# MicroRNAs Modulate the Dynamics of the NF-κB Signaling Pathway

**DOI:** 10.1371/journal.pone.0027774

**Published:** 2011-11-17

**Authors:** Candida Vaz, Arvind Singh Mer, Alok Bhattacharya, Ramakrishna Ramaswamy

**Affiliations:** 1 School of Computational and Integrative Sciences, Jawaharlal Nehru University, New Delhi, India; 2 School of Life Sciences, Jawaharlal Nehru University, New Delhi, India; 3 School of Physical Sciences, Jawaharlal Nehru University, New Delhi, India; Niels Bohr Institute, Denmark

## Abstract

**Background:**

NF-κB, a major transcription factor involved in mammalian inflammatory signaling, is primarily involved in regulation of response to inflammatory cytokines and pathogens. Its levels are tightly regulated since uncontrolled inflammatory response can cause serious diseases. Mathematical models have been useful in revealing the underlying mechanisms, the dynamics, and other aspects of regulation in NF-κB signaling. The recognition that miRNAs are important regulators of gene expression, and that a number of miRNAs target different components of the NF-κB network, motivate the incorporation of miRNA regulated steps in existing mathematical models to help understand the quantitative aspects of miRNA mediated regulation.

**Methodology/Principal findings:**

In this study, two separate scenarios of miRNA regulation within an existing model are considered. In the first, miRNAs target adaptor proteins involved in the synthesis of IKK that serves as the NF-κB activator. In the second, miRNAs target different isoforms of IκB that act as NF-κB inhibitors. Simulations are carried out under two different conditions: when all three isoforms of IκB are present (wild type), and when only one isoform (IκBα) is present (knockout type). In both scenarios, oscillations in the NF-κB levels are observed and are found to be dependent on the levels of miRNAs.

**Conclusions/Significance:**

Computational modeling can provide fresh insights into intricate regulatory processes. The introduction of miRNAs affects the dynamics of the NF-κB signaling pathway in a manner that depends on the role of the target. This “fine-tuning” property of miRNAs helps to keep the system in check and prevents it from becoming uncontrolled. The results are consistent with earlier experimental findings.

## Introduction

**Figure 1:**
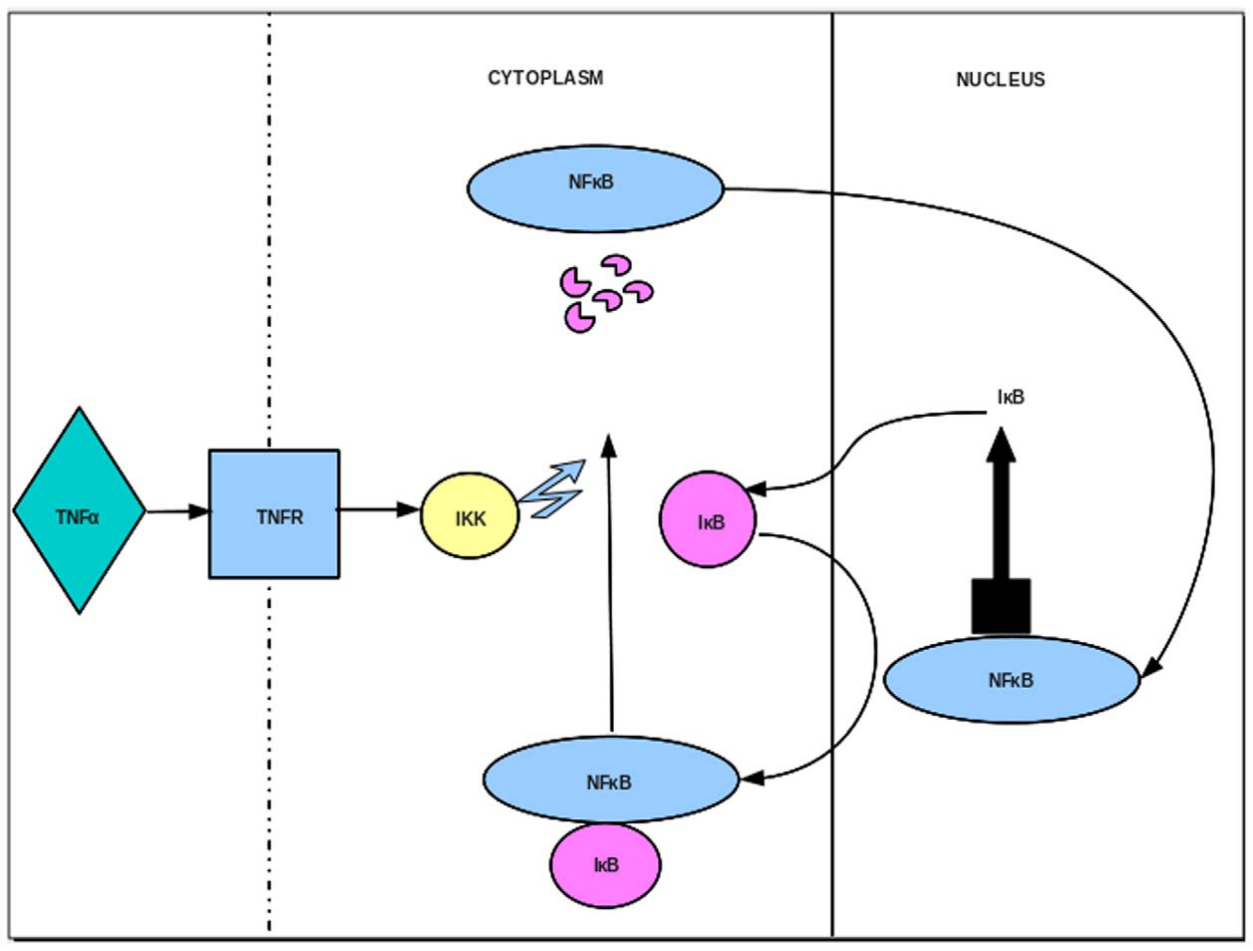
TNFα signaling to NF-κB. NF-κB is held in the latent state in the cytoplasm by its inhibitor IκB. When TNFα binds to the TNF receptor (TNFR), IκB kinase (IKK) gets activated and phosphorylates the inhibitor of NF-κB (IκB) that is subsequently ubiquitinated and degraded. The free NF-κB enters the nucleus where it regulates the transcription of several genes. Among them is the gene for IκB that is up regulated by NF-κB. The synthesized IκB, on binding to NF-κB, promotes its export to the cytoplasm and holds it in latent state thus forming a negative feedback loop.

NF-κB plays a central role in inflammation and immune response [1]. In the unstimulated state it is held inactive by the IκB protein, while cellular stimulation with inflammatory agents results in production of IKK. This mediates phosphorylation, ubiquitination and proteolysis of the IκB resulting in activation of NF-κB and accumulation in the nucleus. Activated NF-κB is a transcription factor that can bind the κB elements in target gene promoters and regulate proinflammatory and immune response related genes [2]. Since misregulation of its levels can cause inflammatory diseases and even cancer [1], the regulation of NF-κB levels is an important mechanism by which development and differentiation of the cells of the immune system are achieved (Figure 1).

The NF-κB pathway is regulated by a number of different mechanisms, such as proteasome mediated degradation, transcription regulation and phosphorylation. Two NF-κB proteins p105 and p100 have long C-terminal domains that inhibit their activity. On receiving appropriate signals active molecules are generated by proteasome mediated cleavage [3], [4]. In canonical signaling, p105 is processed into active p50 but is held as a heterodimer (with p65 or with c-Rel) by its interaction with the IκB proteins. These proteins are phosphorylated by the IKK complex which leads to their degradation mediated by proteasomes, causing release of p50. p50 then moves into the nucleus and activates transcription [5]. In non canonical signaling, p100 is processed into active p52 through activation involving the kinase NIK and IKKα mediated phosphorylation [6].

The canonical pathway is associated with inflammation and innate immune response through stimulation of tumor necrosis factor receptor (TNF) and Toll like receptor (TLR), while the non-canonical pathway is associated with adaptive immunity, secondary lymphoid organs and B cell maturation, and is activated by receptors such as LTβR, BAFF-R, CD40, and RANK [5]. Since TNFα can also activate p100, a potential crosstalk also exists between the two pathways [4]. Indeed it is believed that there is an integrated signaling system involving NF-κB in different cells [4].

The static representation of a pathway cannot capture the dynamics of regulation at a molecular level. Here mathematical models that can quantitatively describe the temporal changes in both space and time and which have been refined by experimental observations [7] have helped in developing a better understanding of NF-κB signaling. Both stochastic and deterministic models have been described in the literature and have been aimed at understanding the mechanism of NF-κB activation through stimulus-induced degradation of IκB [8], the different functions of the three isoforms (IκBα, IκBβ, IκBε) and their roles in different NF-κB dynamics [9], [10].

A major finding is the importance of IκBα (induced by NF-κB) in providing a negative feedback leading to oscillations in NF-κB levels in cells lacking other isoforms. The role of IκBβ and IκBε is to dampen the oscillations and when all the three isoforms are present in the cell, the NF-κB response is biphasic [7].

Extracellular feedback loops arise in response to stimulation by LPS (lipopolysaccharide) which activates two intracellular pathways that branch at the receptor level, dependent on MyD88 and Trif [11]. The NF-κB activity is normally steady when both are present but becomes oscillatory when either of the two is down-regulated since both the pathways have the same activation kinetics but have a 30 min gap in their activation time, causing their oscillations to be out of phase. The sensitivity of NF-κB dynamics to the timing and duration of IKK activity is also demonstrated both by experimentation and modeling [12]. A surge in IKK level resulting from TNFα stimulation generates a transient NF-κB response, whereas a steady state IKK profile resulting from LPS stimulation generates a sustained level of NF-κB activity. Different IKK profiles cause activation of different genes in response to TNFα and LPS stimulation [13].

Ihekwaba *et al* (2004) [14] constructed a NF-κB model containing 64 parameters and 26 variables to which they applied sensitivity analysis. Of these only eight reactions that involved either IKK or IκBα as one of the components exerted a major control on NF-κB oscillations. Hayot and Jayaprakash (2006) [15] investigated cell-to-cell variability using a simplified version of the Hoffman *et al* model [9]. They considered cellular NF-κB and IKK as external parameters, and found intrinsic fluctuations to be insignificant when these were fixed in a model with high level of transcription. The extrinsic fluctuations, such as initial amount of cellular NF-κB and amount of activated IKK were found to be very significant.

Krishna *et al* (2006) [16] address two important questions regarding NF-κB dynamics, on how the network produces oscillations and how these oscillations are used to differentiate among different stimuli and send specific signals to the downstream genes. A small core network (three variable model) has been studied and shown to produce “spiky oscillations” in nuclear NF-κB concentrations. These spiky oscillations are robust to changes in the parameters and are associated with an increased sensitivity of the system to IKK, which is essential for differential regulation of downstream genes.

MicroRNAs are a class of small RNAs involved in post-transcriptional regulation [17]. They are typically about 22 nucleotides in length and are encoded from hairpin shaped transcripts. In metazoans, they control gene expression and many important cellular processes through translational repression and exonucleolytic cleavage of their target mRNAs, by binding to their 3′UTR. Similar to the TF's (transcription factors) miRNAs are trans-acting factors that exert their activity through composite cis-regulatory elements that are encoded within the genomic DNA, and act in a combinatorial manner and cooperatively on their targets [18]. Since miRNAs are tightly linked to TF's their expression patterns are controlled by TF's and vice versa. Cellular regulation is likely to be a network involved in decision making and miRNAs may be required as switches and fine tuners [19], [20].

Recent studies have shown that miRNAs are also involved in regulation of both innate and acquired immunity, particular via the NF-κB pathway [21], [22]. Specifically, miR146a, miR155, miR-125b, miR-9 and miR-29 are known to be involved at different steps in the NF-κB pathway. For instance, while the expression of miR146a is dependent on NF-κB, it also regulates genes that are involved in the NF-κB network [23], [24]. miR-9 is downregulated in ovarian cancers as compared to the normal tissues, and it is also important in inhibiting ovarian cancer by targeting the NF-κB1 mRNA [25]. In myoblasts, expression of miR-29 is repressed by NF-κB through YY1 and polycomb proteins. It serves as a tumor suppressor, since reconstitution of the miRNA inhibits tumor growth and promotes differentiation [26]. Another instance of such regulation comes from the observation that a cluster of 14 miRNAs from the virus KSHV genome regulates the NF-κB pathway by reducing expression of IκBα protein. Deletion of this cluster from the KSHV genome significantly enhances viral lytic replication as a result of reduced NF-κB activity whereas its expression has an opposite effect. This suggests that the KSHV miRNAs are crucial in regulation of viral latency and lytic replication by manipulation of the host survival pathway [27].

A recent review [28] highlights the convergence of miRNAs and NF-κB signalling. The functions of a handful of miRNAs such as miR-146, miR-155, miR-181b, miR-21, miR-301a in the NF-κB system have been discussed in detail, with exclusive emphasis on the feedback loops they are involved in. The miR-146 is induced by NF-κB and in turn negatively regulates IRAK1/TRAF6, constituting a negative feedback loop. MiR-155 is also an NF-κB transactivational target and is involved in a negative feedback loop through regulation of IKK. miR-181b (activated by STAT3), has recently been identified as involved in a positive feedback loop by inhibiting CYLD (negative regulator of NF-κB), which in turn causes increased NF-κB, that completes the loop by leading to STAT3 activation. miR-21 and miR-301 are also known to be involved in a positive feed back loop, by inhibiting genes (PTEN and NKRF respectively) that are negative regulators of NF-κB, and in turn, on activation of NF-κB, themselves get transcribed

Thus a range of studies have clearly shown the importance of miRNAs in the regulation of the NF-κB network. In this work an attempt is made to understand the role of miRNAs in NF-κB system using mathematical models. Two different targets of miRNAs, IKK and IκB's are considered: these are known to be the most important components that reflect the dominant dynamics of the pathway [14], [15]. Our results clearly show that miRNAs can indeed change the dynamic properties of the NF-κB network in a manner that is consistent with that observed during immune response.

## Results

### Scenario 1: NF-κB induced miRNAs target the adaptor proteins needed for the production of IKK

A few miRNAs, such as miR-146 (a, b) and miR-155 are known to target and down-regulate adaptor proteins IRAK1 and TRAF6. Down-regulation of the adaptor proteins causes a reduction in the IKK levels that is essential for activating NF-κB. Unregulated inflammatory response due to infections by pathogens is harmful unless controlled through negative regulation and most known attenuation mechanisms of inflammation involve negative transcriptional feedback loops such as IκB and A20. Adding miRNAs to the list of potential negative regulators of inflammation would provide an improved understanding of immune regulation [23]. In order to test this aspect and to understand the mechanisms of participation of miRNAs in the NF-κB system, miRNA regulated steps were included in the existing model, and the adaptors producing IKK were considered as targets of the miRNAs. The model proposed by Hoffman *et al* [9] is shown in Figure 1. Of the three isoforms of IκB, when only IκBα is allowed to be expressed and others are knocked out, NFκB levels oscillate, consistent with the original model, though a biphasic curve is obtained with all three isoforms of IκB.

**Figure 2:**
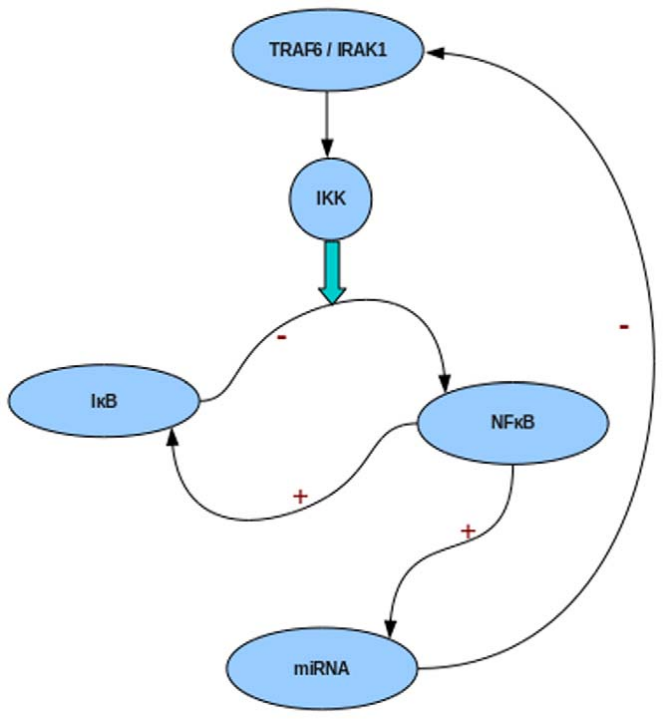
Scenario 1. IRAK1 and TRAF6 are adaptor proteins producing IKK that frees the NF-κB from inhibition by IκB. The NF-κB induced miRNA, targets the IRAK1/TRAF6 mRNA. The sign “+” indicates activation whereas the sign “-“indicates repression.

The NF-κB induced miRNA negatively regulates the adapter proteins TRAF6/IRAK1, thereby controlling IKK levels (Figure 2). The set of relations used to describe the different steps involving miRNA is given below. Briefly, once the pre-miRNA is synthesized, Dicer processing of the pre-miRNA occurs, involving the unwinding of the duplex followed by incorporation of only one strand into the miRISC, the multiprotein complex that uses the miRNA as a template for recognizing the target (complementary) mRNA.

MiRNAs associate with their targets by base-pair complementarity. In plants, miRNAs show nearly perfect complementarity to their target sequences generally located in the coding regions or 3′ untranslated regions (3′ UTR) of the mRNA. The perfect base pairing triggers mRNA degradation. In animals, miRNAs regulate gene expression by base pairing imperfectly to the 3′ UTRs of the target mRNA and inhibiting protein synthesis or causing mRNA degradation. One major feature of animal miRNA-mRNA interactions are the contiguous pairing in the miRNA 5′ seed region (commonly positions 2–8) and lack of complementarity in the central region. The deciding factor between miRNA induced mRNA degradation and miRNA induced translational repression, is the extent of complementarity and presence of central matches between the miRNA and its target [29], [30].

The repressed mRNAs accumulate in P-bodies or GW bodies [31], that serve as sites of translational repression and mRNA decay and are enriched in factors that are involved in these pathways. Most importantly, this process can be reversed, indicating that P-bodies can serve as temporary storage sites for mRNAs not participating in protein synthesis. This reversal of repression causing the release of the repressed mRNA occurs under specific conditions related to environmental stress [32] or developmental cues [33].

Based on the mechanisms of miRNA action two cases were considered [34]: Case 1: Degradation of the target mRNA in the RISC (non-reversible miRNA mediated repression), Case 2: Regeneration of the target mRNA from the RISC (reversible miRNA mediated repression).
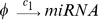
(1)

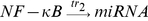
(2)

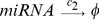
(3)

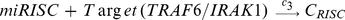
(4)

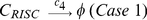
(5)

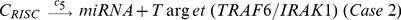
(6)

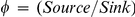



Equations 1, 2 imply the introduction of miRNA into the system through constitutive transcription (c_1_) and also through NF-κB induced transcription (tr_2_). The equation 3 represents the degradation of miRNA at a constant rate (c_2_). Equation 4 (c_3_) is a simplistic form of representing the entire process right from the incorporation of a single stranded mature miRNA into the RISC to give miRISC, which binds to the target mRNA forming a miRNA:mRNA duplex in the RISC (C_RISC_). Equation 5, governed by the rate constant c_4_ denotes the degradation of the target mRNA in the RISC, whereas the Equation 6, with the rate constant c_5_ denotes an alternative channel that may be operative in some cases where due to certain environmental or developmental conditions the RISC releases the target mRNA back to the pool. The ratio between Equation 5 and 6 depends on the extent of complementarity between the miRNA and its target sequence as mentioned before.

#### A. Wild Type system

**Figure 3:**
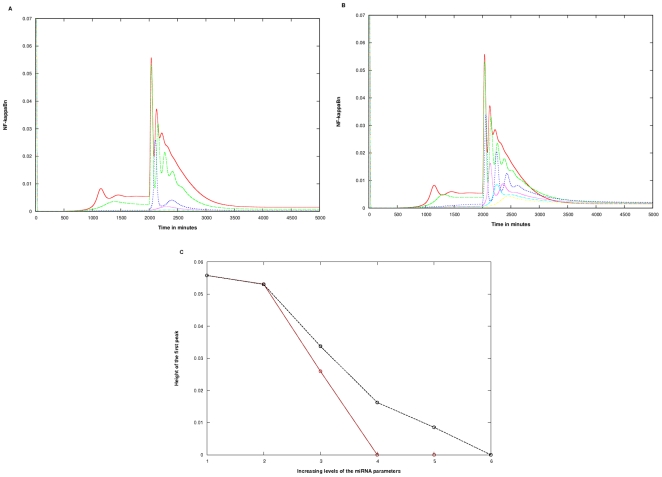
Effect of miRNA targeting the IRAK1/TRAF6 complex in the wild type. The miRNA parameters c_1_, c_3_ and c_4_ or c_5_ were varied, keeping c_2_ at 0.00048 min^−1.^ A) Case 1 B) Case 2. The colored lines in the figure represent the effect at varying levels of the three miRNA parameters. The parameter values in parenthesis are c_1_, c_3_, c_4_/c_5_ respectively: Red (0.00, 0.000, 0.000) Green (0.01, 0.001, 0.001), Blue (0.03, 0.003, 0.003), Pink (0.05, 0.005, 0.005), Cyan (0.07, 0.007, 0.007), Yellow (0.10, 0.010, 0.010). In the y-axis title “NF-kappaBn”, the “n” stands for nuclear. The Quantification was done in terms of C) Alteration in the height of the first peak. This Figure section is derived from sections A, B. The x-axis signifies the 6 levels of variation of the three parameters as given in sections A, B. The Brown and Black lines represent Cases 1 and 2 respectively.

The biphasic plot characteristic of the wild type becomes oscillatory with increase in the values of all the parameters: c_1_, c_3_, and c_4_ or c_5_. Here, c_1_, c_3_ describe the production (c_1_) (varied from 0.01 to 0.1 min^−1^) and binding (c_3_) (varied from 0.001 to 0.01 min^−1^) rates respectively. For Case 1, c_4_ (varied from 0.001 to 0.01 min^−1^) represents the mRNA degradation rate in the RISC; for Case 2, c_5_ (varied from 0.001 to 0.01 min^−1^) denotes the mRNA regeneration rate from the RISC. Moreover, as the levels of the parameters were increased, the frequencies as well as the amplitude of the oscillations were found to decrease significantly (Figure 3A, 3B). However, for Case 2 (Figure 3B), the changes in oscillatory behavior, such as decrease in amplitude, was observed to be less dramatic than Case 1 (Figure 3A) on increasing the rate constants. Since the numbers of oscillations produced on introduction of miRNAs were too few to be quantified, the numbers of peaks were counted for indicating the change in frequency; also decrease in the height of the first peak, was taken as a measure of the negative potential of the miRNAs in the system. The minimum level of the parameters did not show much change among the two cases. The number of peaks at increasing levels of the three miRNA related parameters (as given in the figure legend), were two (blue line), one (pink line), none (cyan and yellow) as compared to three, two and one at the same levels in Case 2 (Figure 3A, 3B).

The height of the first peak, a measure of the peak NFκB concentration, decreased much more sharply in Case 1 than in Case 2. The average decrease of the peak for Case 1 was twice that for Case 2 (Figure 3C).

The contribution of each of the parameters in the Wild type was determined by varying one and keeping the other two constant. Variation of the c_1_ parameter (from 0.01 to 1.00 min^−1^, keeping c_3_ at 0.001 min^−1^ and c_4/_c_5_ at 0.001 min^−1^, and variation of the c_3_ parameter (from 0.001 to 0.01 min^−1^), keeping c_1_ and c_4/_c_5_ at 0.01 min^−1^ and at 0.001 min^−1^ respectively, brought about a decrease in the frequency and amplitude (Figures S1A, B, C, D). Case 1 showed a sharper decrease as compared to Case 2 (Figures S2A, B). Variation in the c_4_ and c_5_ parameter from 0.001 to 0.01 min^−1^, keeping c_1_ at 0.01 min^−1^ and c_3_ at 0.01 min^−1^ in Case 1 and Case 2 respectively, showed an interesting behavior (Figures S1E, F). While both cases showed a sharp decrease in the height of the first peak (from 0.056 to 0.023 in Case 1 and to 0.028 in Case 2) when miRNA was introduced in the model, an increase in c_4_ did not have any effect on the height in Case 1 beyond that. The Case 2, however, showed an increase in peak height with an increase in c_5_ (average slope being 0.94). This is presumably because a higher c_5_ in this case results in higher levels of translatable mRNA being released from the RISC complex. This explains the reason behind the prolonged oscillations and marginal changes in the amplitude in Case 2 causing the overall differences in the behaviour of the system in the two cases (Figure S2C).

#### B. Knockout Type System

**Figure 4:**
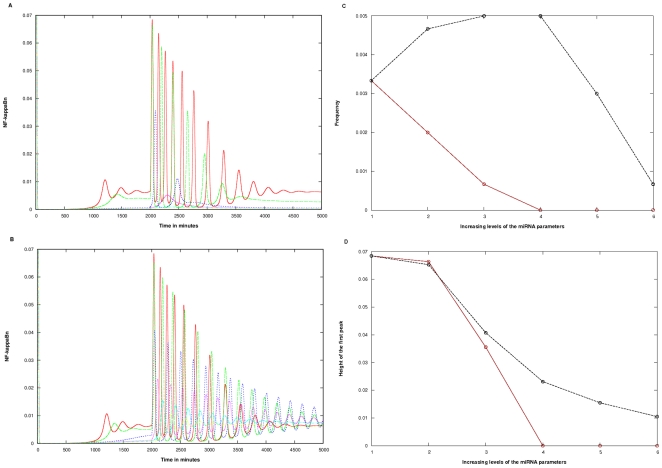
Effect of miRNA targeting the IRAK1/TRAF6 complex in the knockout type. The miRNA parameters c_1_, c_3_ and c_4_ or c_5_ were varied, keeping c_2_ at 0.00048 min^−1^. A) Case 1 B) Case 2. The colored lines in the figure represent the effect at varying levels of the three miRNA parameters. The parameter values in parenthesis are c_1_, c_3_, c_4_/c_5_ respectively: Red (0.00, 0.000, 0.000), Green (0.01, 0.001, 0.001), Blue (0.03, 0.003, 0.003), Pink (0.05, 0.005, 0.005), Cyan (0.07, 0.007, 0.007), Yellow (0.10, 0.010, 0.010). In the y-axis title “NF-kappaBn”, the “n” stands for nuclear. The Quantification was done in terms of: C) Alteration in frequency D) Alteration in the height of the first peak. Figure sections C, D are derived from sections A, B. The x-axis signifies the 6 levels of variation of the three parameters as given in sections A, B. The Brown and Black lines represent Cases 1 and 2 respectively.

Introduction of miRNAs in the system wherein the IκBβ and IκBε isoforms were knocked down leaving only the IκBα isoform, reduced the frequency as well as the amplitude of the pre-existing oscillations. The parameters were increased in a similar manner as done for the wild type system. A prolonged response was also noticed in this system, when mRNA (target) and miRNAs were allowed back to the pool from the RISC (Case 2) (Figure 4B) in comparison to the Case 1 (Figure 4A).

The minimum level of the parameters did not show much change among the two cases. The decrease in the frequency and height of the first peak was taken as a measure of the negative potential of the miRNA in the system.

The frequency showed different patterns for the two cases (Figure 4C). In Case 1, the frequency decreased steadily (slope = 0.001) as the parameter levels were increased, up to level 4, after which the oscillations stopped. In Case 2, however, the frequency stayed nearly constant (at around 0.005, which was actually higher than 0.0033, the frequency without the introduction of miRNA into the model) till the parameters were increased to level 4, after which it steadily decreased (slope = 0.00215).

Similarly, as shown in Figure 4D, while the height of the first peak fell sharply (and linearly) in Case 1, with a decrease of about (0.03375) units per increase of parameter level before vanishing at level 4, this fall was non-linear, and shallower for Case 2 (an average fall of (0.01425).

The contribution of each of the miRNA parameters in the Knockout type was determined by varying one and keeping the other two constant, as described in the previous section for the Wild type. Variation of the c_1_ parameter (from 0.01 to 1.00 min^−1^), keeping c_3_ at 0.001 min^−1^ and c_4_/c_5_ at 0.001 min^−1^ and variation in the c_3_ parameter (from 0.001 to 0.01 min^−1^), keeping c_1_ and c_4/_c_5_ at 0.01 min^−1^ and at 0.001 min^−1^ respectively, brought about a sharp decrease in frequency and amplitude in both the cases. (See Figures S3A, B, C, D and S4A, B, C, D)

Variation of the c_4_ parameter and c_5_ parameter (from 0.001 to 0.01 min^−1^) in Case 1 and 2 respectively, keeping c_1_ at 0.01 min^−1^ and c_3_ at 0.01 min^−1^, showed a striking behavior (Figures S3E, F). While the frequency reduced to 0.0007 in Case 1, it increased from 0.002 to 0.005 in Case 2 as c_5_ was increased up to 0.003, staying constant thereafter (Figure S4E). The height of the first peak also showed an increase (slope = 0.5) in Case 2, while it remained at 0.032 in Case 1, showing no variation as c_4_ was varied between 0.001 and 0.01. (See Figure S4F).

The findings can therefore be restated: the role of c_1_ and c_3_ parameters is to reduce the level of NFκB, in both the cases. The parameter c_5_ is the one responsible for the prolonged oscillations and higher amplitude and frequency in Case 2 as compared to Case 1.

### Scenario 2: miRNAs target the IκB isoforms

The IκB isoforms have important roles as inhibitors of NF-κB, holding it in the latent state. It has been seen that some miRNAs or miRNA clusters target these IκB's, and are therefore involved in regulating the NF-κB pathway. Deletion of these miRNAs from the Kaposi's sarcoma-associated herpes virus genome (KSHV) enhances viral lytic replication as a result of reduced NF-κB activity. Thus, KSHV encodes miRNAs to control viral replication by activating the NF-κB pathway. These results illustrate an important role for KSHV miRNAs in regulating viral latency and lytic replication by manipulating a host survival pathway [27].

**Figure 5:**
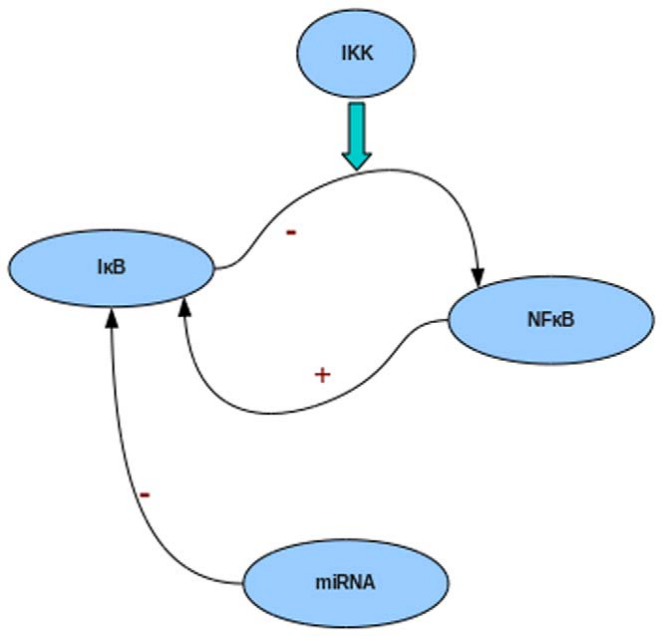
Scenario 2. IKK frees NF-κB from inhibition by IκB. The miRNA targets the inhibitors of NF-κB (IκB). The sign “+” indicates activation whereas the sign “−” indicates repression.



Here miRNA negatively regulates the IκB's that hold the NF-κB in latent state (Figure 5). The set of relations used to describe different steps involving miRNA is described below. Here again two Cases of miRNA action were considered: Case 1: Degradation of the target mRNA in the RISC, Case 2: Regeneration of the target mRNA from the RISC.

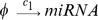
(7)

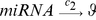
(8)

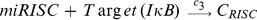
(9)

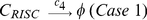
(10)

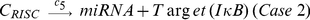
(11)

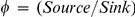



Equation 7 represents the introduction of miRNA into the system through constitutive transcription (c_1_) only. The equation 8 represents the degradation of miRNA at a constant rate (c_2_). Equation 9 (c_3_) is a simplistic form of representing the entire process right from the incorporation of a single stranded mature miRNA into the RISC to give miRISC, which binds to the target mRNA (here the IκB's) forming a miRNA:mRNA duplex in the RISC (C_RISC_). Equation 10, governed by the rate constant c_4_ denotes the degradation of the target mRNA in the RISC, whereas the Equation 11, with the rate constant c_5_ denotes an alternative channel that may be operative in some cases where due to certain environmental conditions the RISC releases the target mRNA back to the pool.

Individual, as well as combinations of IκB's were analyzed as miRNA targets. Striking types of behaviour patterns on increasing the miRNA related parameters such as the production (c_1_) (from 0.01 to 0.1 min^−1^), binding (c_3_) (from 0.001 to 0.01 min^−1^) and RISC degradation rates (c_4_/c_5_) (from 0.0001 to 0.001 min^−1^) all together, keeping c_2_ constant at 0.00048 min^−1^ were noticed.

#### A) Distortion or no change in the biphasic behavior of the wild type

In the following target assumptions, except for IκBε, the biphasic plot gets distorted making the effect immeasurable. When IκBε is the target, there occurs no change in the dynamics (Figure S5):

Only IκBα as target

Only IκBε, as target

IκBα and IκBβ as target

IκBα and IκBε, as target

All the three IκB's as target

#### B) Conversion from the biphasic to oscillatory behavior

In the following two target assumptions, a drastic change in the behaviour was noticed. Firstly, the initial biphasic became oscillatory similar to the knockout type behaviour. Secondly, with the increase in the miRNA related parameters, the frequency and amplitude of the oscillations increased.

**Figure 6:**
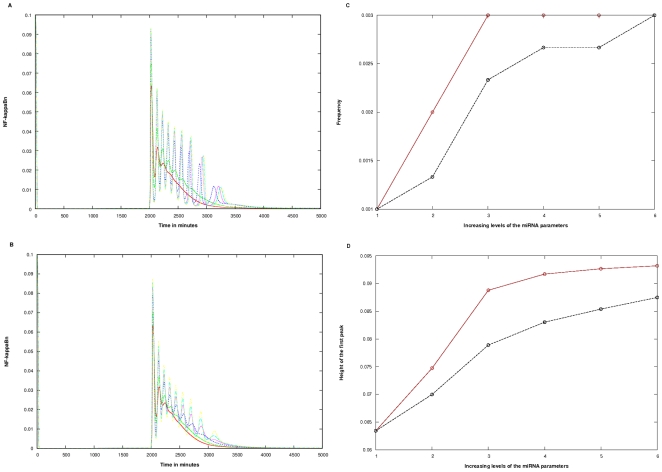
Conversion from wild type to oscillatory type behaviour on incorporation of miRNA targeting only IκBβ. A) Case 1 B) Case 2. The colored lines in the figure represent the effect at varying levels of the three miRNA parameters. The parameter values in parenthesis are c_1_, c_3_, c_4_/c_5_ respectively: Red (0.00, 0.000, 0.000), Green (0.01, 0.001, 0.001), Blue (0.03, 0.003, 0.003), Pink (0.05, 0.005, 0.005), Cyan (0.07, 0.007, 0.007), Yellow (0.10, 0.010, 0.010). In the y-axis title “NF-kappaBn”, the “n” stands for nuclear. The Quantification was done in terms of: C) Alteration in the frequency D) Alteration in the height of the first peak. Figure sections C, D are derived from sections A, B. The x-axis signifies the 6 levels of variation of the three parameters as given in sections A, B. The Brown and Black lines represent Cases 1 and 2 respectively.

When only IκBβ was considered as the target (Figure 6A, 6B): The positive role of the miRNA was measured in terms of both, the frequency and the height of the first peak. The frequency increased from 0.002 to 0.003 in Case 1 and 0.001 to 0.003 in Case 2 (Figure 6C). The height of the first peak increased from 0.075 to 0.093 in Case 1 and 0.070 to 0.087 in Case 2 when only IκBβ was the target (Figure 6D). Figure 6C shows that the frequency increased faster in Case 1, increasing from 0.002 to 0.003 as the parameters was increased to level 3, keeping constant at higher levels; Case 2 showed average increases of 0.000425 per level as miRNA parameter levels were varied between 2 and 6. The height of the first peak increased relatively similarly (slopes 0.0045 and 0.0046 for Cases 1 and 2, respectively) between parameter levels 2 and 6 (Figure 6D).

**Figure 7:**
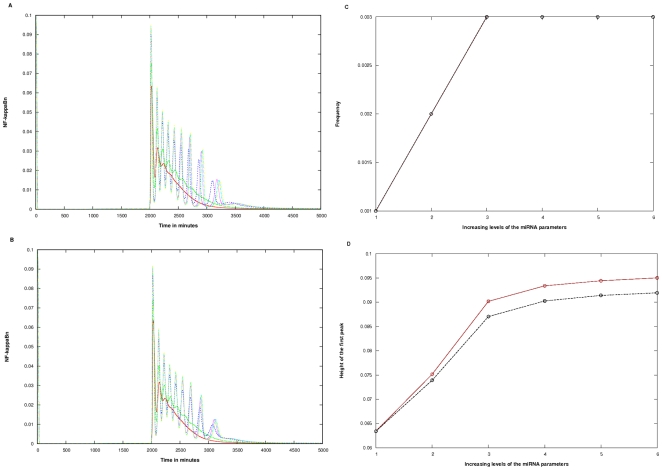
Conversion from wild type to oscillatory type behaviour on incorporation of miRNA targeting both IκBβ and IκBε. A) Case 1 B) Case 2. The colored lines in the figure represent the effect at varying levels of the three miRNA parameters. The parameter values in parenthesis are c_1_, c_3_, c_4_/c_5_ respectively: Red (0.00, 0.000, 0.0000), Green (0.01, 0.001, 0.0001), Blue (0.03, 0.003, 0.0003), Pink (0.05, 0.005, 0.0005), Cyan (0.07, 0.007, 0.0007), Yellow (0.10, 0.010, 0.0010). In the y-axis title “NF-kappaBn”, the “n” stands for nuclear. The Quantification was done in terms of: C) Alteration in the frequency D) Alteration in the height of the first peak. Figure sections C, D are derived from sections A, B. The x-axis signifies the 6 levels of variation of the three parameters as given in sections A, B. The Brown and Black lines represent Cases 1 and 2 respectively.

When both IκBβ and IκBε were considered as targets (Figure 7A, 7B): In contrast, the level of enhancement of frequency when both IκBβ and IκBε, were targeted (Figure 7C) was not discernible (both increased from 0.001 to 0.003 as the parameter levels were increased from 2 to 6). The height of the first peak also increased almost exactly in same manner for the two cases (Figure 7D), though Case 2 seemed to show slower increase at higher levels of parameters (slope for Case 2 is 0.009 between levels 4 and 6, while it is 0.01 for Case 1).

## Discussion

The importance of miRNAs in gene regulatory networks is becoming clearer with the discovery of a large number of miRNAs that target almost the entire transcriptome. miRNAs alter the network features of gene regulatory modules by targeting key molecules and thus control many of the cellular decision making systems [35]. The quantitative features and dynamics of the effect of miRNAs in pathways and networks have not however been adequately addressed. In this paper we have studied miRNA mediated dynamics of a regulatory system through computational modeling and simulation. The NF-κB pathway was chosen in part since it has been extensively studied both experimentally and via modeling and further, miRNAs are known to affect the regulation. The existing mathematical model was suitably modified by incorporation of miRNAs that target two major entities (IKK, IκB's) involved in the most sensitive dynamics of the pathway. The two conditions were studied by considering two separate scenarios.

miRNAs target the adaptor proteins involved in IKK synthesis [23]. There are three isoforms of IκB's and oscillations are observed only when IκBα is present while the others are knocked out. Our simulations were for both wild as well as the knockout type. Interestingly, oscillations in wild type situation were observed when miRNAs target the adaptor proteins that are involved in producing IKK. The miRNAs caused the biphasic behaviour in the wild type to become oscillatory, with a subsequent decrease in the amplitude and frequency of oscillations. In the knockout system, the amplitude and frequency of oscillations that are already present showed a decrease, suggesting that IKK is an important entity in controlling the dynamics of NF-κB system. This has also been seen in a number of experimental systems: for instance *Helicobacter pylori* infection is controlled by miR-155 that targets IKK directly [36].

Reduction in the level of IKK due to miRNAs results in the decrease in NF-κB levels. Since in this scenario miRNAs are also induced by NF-κB, their levels reduce when NF-κB levels fall, causing IKK levels to increase. This in turn causes NF-κB levels to rise. The involvement of miRNAs in a negative feedback loop brings about the conversion from the characteristic biphasic to oscillatory behaviour. On further reduction in the IKK levels as miRNA levels are increased there is less activated NF-κB, and this leads to a decrease in the frequency and amplitude of the oscillations. In this case miRNAs act as negative regulators of the pathway, ensuring that the immune response does not go out of control.

In the second scenario, IκB's were considered as targets for miRNAs, an instance of which is the case of KSHV miRNAs that target IκBα [27]. When IκBβ or both IκBβ and IκBε were targeted by miRNAs the dynamics became oscillatory, but with an *increase* in frequency and amplitude of oscillations as the rates of miRNA participation increased. Thus here miRNAs act as positive regulators of the pathway. This is somewhat similar to the studies wherein the isoforms are knocked out by external aids; miRNAs perform the same function naturally within the system.

All the three isoforms have different functions and IκBα is the one responsible for the oscillations owing to the negative feedback loop it is involved in. The other two forms play a role in masking these oscillations. The role of IκBε is not clear since when it is targeted there is no change at all, indicating that it may have an indirect role or it may act only in combination with other isoforms IκBβ and IκBα.

The results are consistent with earlier experimental findings. IκBα is involved in a negative auto regulatory loop reducing the activity of NF-κB. The reaction corresponding to this inducible transcription is also the most important and sensitive of all the reactions in the pathway, and therefore when only the IκBα isoform is present and the other two are removed, the dynamics becomes oscillatory. The importance of IκBα (4 out of the 7 cases) is clear from the fact that when it is targeted the biphasic plot gets distorted and the single peak is also removed (See Figure S5).

Thus, in this study we show that oscillations also result when miRNAs are introduced in the system. Depending on the nature and role of the target protein in the pathway, the effect of the miRNA can be to enhance or attenuate NF-κB levels.

There are experimental observations that have verified the involvement of miRNAs in these steps. miRNA induced oscillatory behaviour can be a useful mechanism in controlling inflammatory response induced by infection and other agents, as it helps to dampen the response and contain infection. Uncontrolled response can cause serious damage to the organism and miRNAs can therefore serve to keep them in check. The models and results presented here can be a basis of understanding inflammatory response under different situations and help in developing better therapeutics. Our study is consistent with the current understanding of the NF-κB pathway. The approach used in this study can also be useful in studying role of miRNAs in different regulatory and control circuits.

## Materials and Methods

We consider a model of the NF-κB pathway [9] that comprises 64 reactions and involves 24 species. miRNA related processes were then included, and their time evolution was studied within a deterministic formalism, namely by deriving the appropriate set of coupled differential equations. Codes were written in C and Perl. Parameters related to the miRNA regulation were chosen to correspond to experimentally estimated rates. Since intracellular miRNA steady state levels result from not only the synthesis of new miRNAs but also the degradation of these miRNAs, characterisation of miRNA persistence is indispensable. For the miRNA degradation rate the estimate given in a recent work by Khanin and Vinciotti was used [37]. They developed a kinetic model of post-transcriptional regulation by miRNAs that made an experimentally verifiable prediction of miR-124a decay rate (0.024 h^−1^). This decay rate corresponds to a half-life of 29 h with 95% confidence bounds (26 h, 50 h).

In a recent study [38] miRNA decay rates were measured in Mouse embryonic fibroblasts following the loss of Dicer 1 enzymatic activity. The half-life was measured to be 21.6 h. Intracellular miRNA levels were affected by dilution from cellular division, and therefore to calculate the miRNA half-life, independent of cellular division and any residual Dicer 1 activity, the decay rates of a panel of six miRNAs were measured and a mathematical model was established to predict miRNA stability in a non-dividing cell population devoid of any Dicer activity. An average miRNA half life was determined to be 119 h (5 days) [38].

## Supporting Information

Figure S1 **Qualitative effect of miRNA targeting the IRAK1/TRAF6 complex in the wild type.** When only c_1_ is varied, keeping c_3_ at 0.001, c_4_/c_5_ at 0.001: A) Case 1 B) Case 2. The colored lines in the figure represent the effect at varying levels of the c_1_ parameter. The parameter value in the parenthesis is c_1_ only: Red (0.00), Green (0.01), Blue (0.05), Pink (0.10), Cyan (0.50), and Yellow (1.00). When only c_3_ is varied, keeping c_1_ at 0.01, c_4_/c_5_ at 0.001: C) Case 1 D) Case 2. The colored lines in the figure represent the effect at varying levels of the c_3_ parameter. The parameter value in the parenthesis is c_3_ only: Red (0.000), Green (0.001), Blue (0.003), Pink (0.005), Cyan (0.007), and Yellow (0.010). When only c_4_ and c_5_ are varied in Case 1 and Case 2 respectively, keeping c_1_ at 0.01, c_3_ at 0.01: E) Case 1 F) Case 2. The colored lines in the figure represent the effect at varying levels of the c_4_/c_5_ parameter. The parameter value in parenthesis is c_4_/c_5_ only: Red (0.000), Green (0.001), Blue (0.003), Pink (0.005), Cyan (0.007), and Yellow (0.010). In the y-axis title “NF-kappaBn”, the “n” stands for nuclear.
(TIFF)

Figure S2 **Quantification of the effect of miRNA, targeting the IRAK1/TRAF6 complex in the wild type.** Quantification was done in terms of alteration in the height of the first peak. A) When only c_1_ is varied. B) When only c_3_ is varied. C) When only c_4_ and c_5_ are varied in Case 1 and Case 2 respectively. The x-axis signifies the variation of the parameter individually, keeping the other two constant as given in S 1. The Brown and Black lines represent Cases 1 and 2 respectively.
(TIFF)

Figure S3 **Qualitative effect of miRNA targeting the IRAK1/TRAF6 complex in the knockout type.** When only c_1_ is varied, keeping c_3_ at 0.001, c_4_/c_5_ at 0.001: A) Case 1 B) Case 2. The colored lines in the figure represent the effect at varying levels of the c_1_ parameter. The parameter value in the parenthesis is c_1_ only: Red (0.00), Green (0.01), Blue (0.05), Pink (0.10), Cyan (0.50), and Yellow (1.00). When only c_3_ is varied, keeping c_1_ at 0.01, c_4_/c_5_ at 0.001: C) Case 1 D) Case 2. The colored lines in the figure represent the effect at varying levels of the c_3_ parameter. The parameter value in the parenthesis is c_3_ only: Red (0.000), Green (0.001), Blue (0.003), Pink (0.005), Cyan (0.007), and Yellow (0.010). When only c_4_ and c_5_ are varied in Case 1 and Case 2 respectively, keeping c_1_ at 0.01, c_3_ at 0.01: E) Case 1 F) Case 2. The colored lines in the figure represent the effect at varying levels of the c_4/_c_5_ parameter. The parameter value in parenthesis is c_4_/c_5_ only: Red (0.000), Green (0.001), Blue (0.003), Pink (0.005), Cyan (0.007), and Yellow (0.010). In the y-axis title “NF-kappaBn”, the “n” stands for nuclear.
(TIFF)

Figure S4 **Quantification of the effect of miRNA targeting the IRAK1/TRAF6 complex in the knockout type.** When only c_1_ is varied: A) In terms of alteration in frequency B) In terms of alteration in the height of the first peak. When only c_3_ is varied: C) In terms of alteration in frequency D) In terms of alteration in the height of the first peak. When only c_4_ and c_5_ are varied in Case 1 and Case 2 respectively: E) In terms of alteration in frequency F) In terms of alteration in the height of the first peak. The x-axis signifies the variation of the parameter individually, keeping the other two constant as given in S 3. The Brown and Black lines represent Cases 1 and 2 respectively.
(TIFF)

Figure S5 **The biphasic plot gets distorted when the miRNA target is:** Only IκBα: A) Case 1 B) Case 2. Only IκBε: C) Case 1 D) Case 2. Both IκBα and IκBβ: E) Case 1 F) Case 2. Both IκBα and IκBε: G) Case 1 H) Case 2. All the three IκB's (IκBα, IκBβ, IκBε): I) Case 1 J) Case 2. In the y-axis title “NF-kappaBn”, the “n” stands for nuclear.
(TIFF)
