# Supplementary figures and images for: MicroRNAs Modulate the Dynamics of the NF-κB Signaling Pathway
Source: PLoS One. 2011 Nov 17;6(11):e27774. doi: 10.1371/journal.pone.0027774 (PMC3219691; doi:10.1371/journal.pone.0027774)

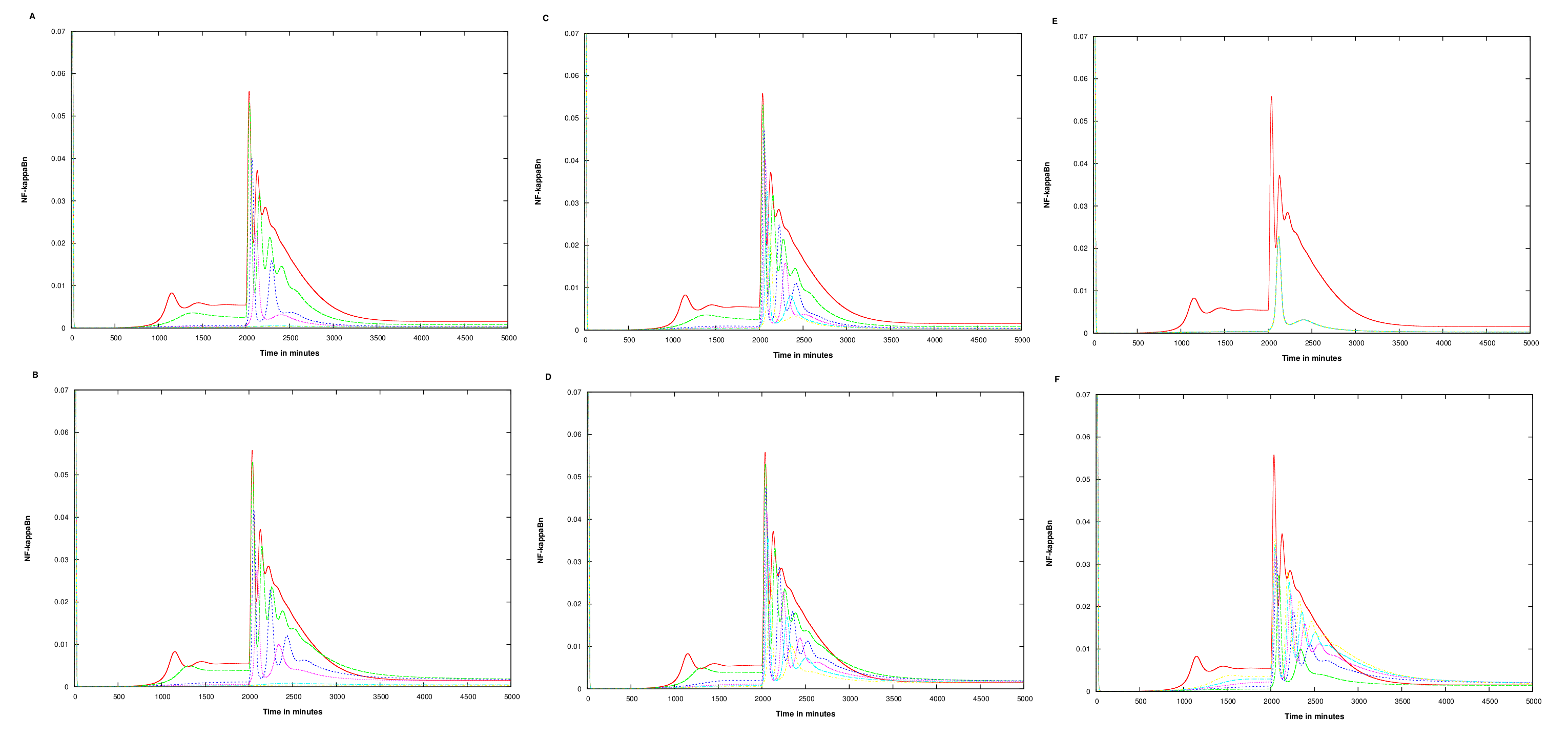

Supplement: Figure S1 — Qualitative effect of miRNA targeting the IRAK1/TRAF6 complex in the wild type. When only c1 is varied, keeping c3 at 0.001, c4/c5 at 0.001: A) Case 1 B) Case 2. The colored lines in the figure represent the effect at varying levels of the c1 parameter. The parameter value in the parenthesis is c1 only: Red (0.00), Green (0.01), Blue (0.05), Pink (0.10), Cyan (0.50), and Yellow (1.00). When only c3 is varied, keeping c1 at 0.01, c4/c5 at 0.001: C) Case 1 D) Case 2. The colored lines in the figure represent the effect at varying levels of the c3 parameter. The parameter value in the parenthesis is c3 only: Red (0.000), Green (0.001), Blue (0.003), Pink (0.005), Cyan (0.007), and Yellow (0.010). When only c4 and c5 are varied in Case 1 and Case 2 respectively, keeping c1 at 0.01, c3 at 0.01: E) Case 1 F) Case 2. The colored lines in the figure represent the effect at varying levels of the c4/c5 parameter. The parameter value in parenthesis is c4/c5 only: Red (0.000), Green (0.001), Blue (0.003), Pink (0.005), Cyan (0.007), and Yellow (0.010). In the y-axis title “NF-kappaBn”, the “n” stands for nuclear. (TIFF) [file pone.0027774.s001.tiff]

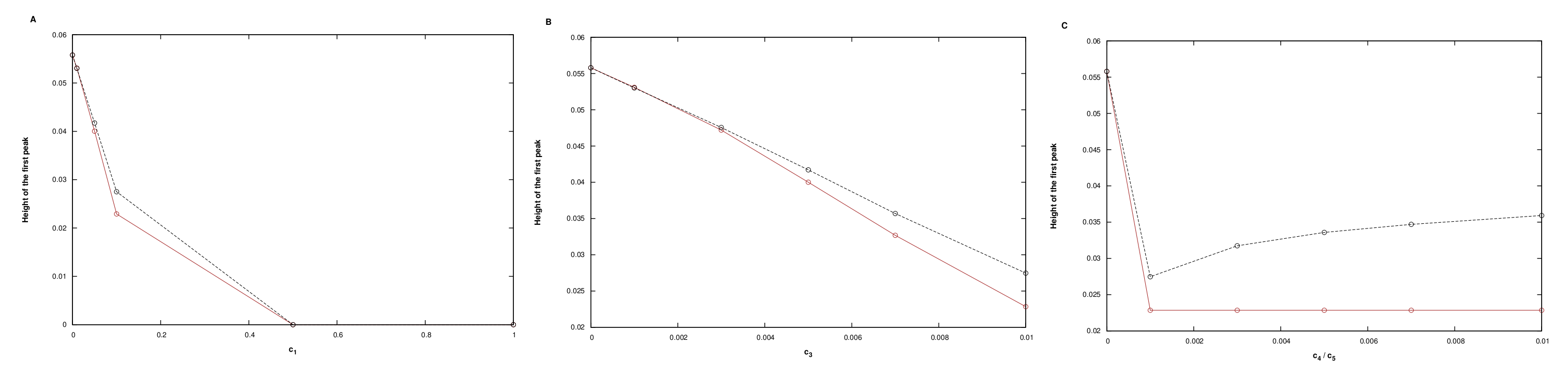

Supplement: Figure S2 — Quantification of the effect of miRNA, targeting the IRAK1/TRAF6 complex in the wild type. Quantification was done in terms of alteration in the height of the first peak. A) When only c1 is varied. B) When only c3 is varied. C) When only c4 and c5 are varied in Case 1 and Case 2 respectively. The x-axis signifies the variation of the parameter individually, keeping the other two constant as given in S 1. The Brown and Black lines represent Cases 1 and 2 respectively. (TIFF) [file pone.0027774.s002.tif]

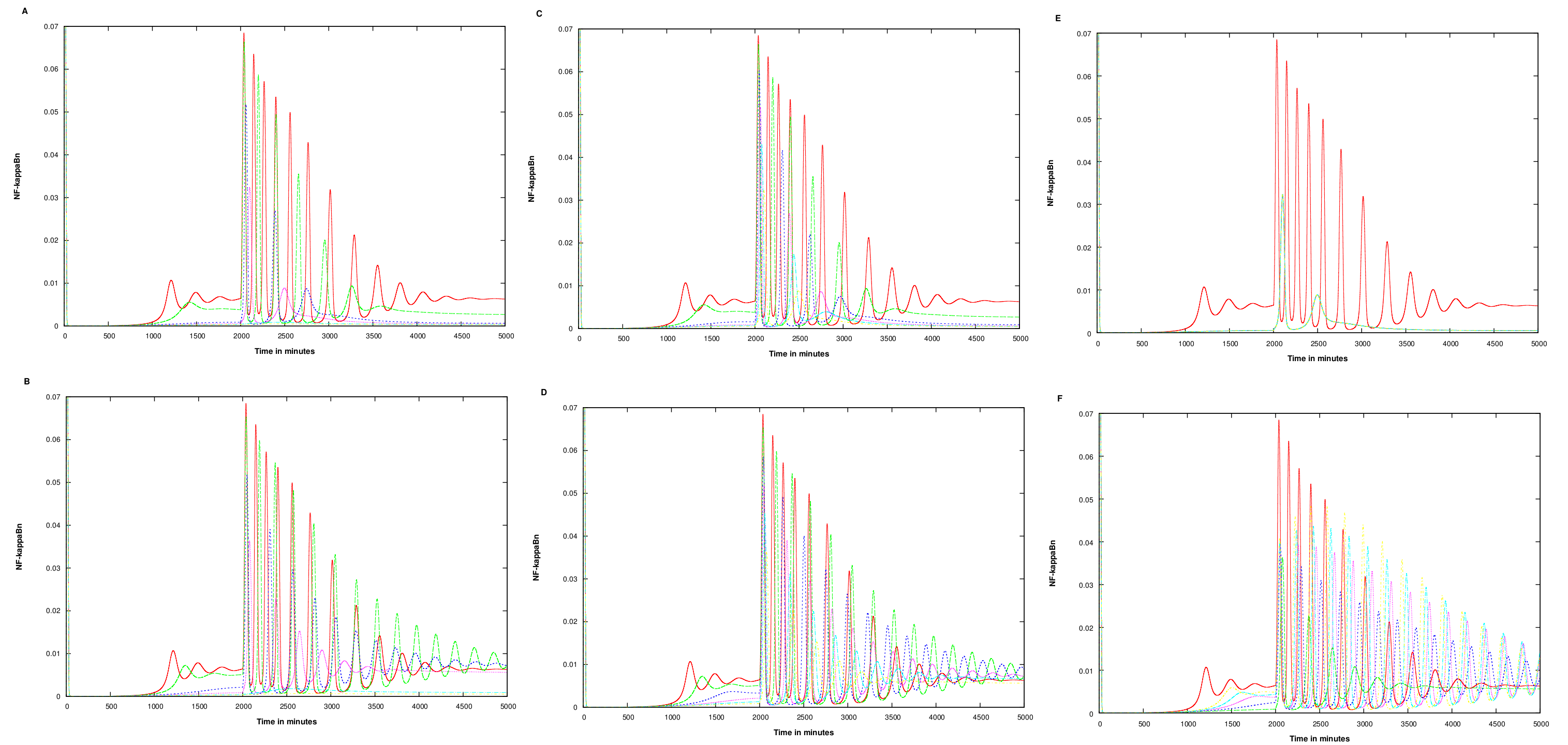

Supplement: Figure S3 — Qualitative effect of miRNA targeting the IRAK1/TRAF6 complex in the knockout type. When only c1 is varied, keeping c3 at 0.001, c4/c5 at 0.001: A) Case 1 B) Case 2. The colored lines in the figure represent the effect at varying levels of the c1 parameter. The parameter value in the parenthesis is c1 only: Red (0.00), Green (0.01), Blue (0.05), Pink (0.10), Cyan (0.50), and Yellow (1.00). When only c3 is varied, keeping c1 at 0.01, c4/c5 at 0.001: C) Case 1 D) Case 2. The colored lines in the figure represent the effect at varying levels of the c3 parameter. The parameter value in the parenthesis is c3 only: Red (0.000), Green (0.001), Blue (0.003), Pink (0.005), Cyan (0.007), and Yellow (0.010). When only c4 and c5 are varied in Case 1 and Case 2 respectively, keeping c1 at 0.01, c3 at 0.01: E) Case 1 F) Case 2. The colored lines in the figure represent the effect at varying levels of the c4/c5 parameter. The parameter value in parenthesis is c4/c5 only: Red (0.000), Green (0.001), Blue (0.003), Pink (0.005), Cyan (0.007), and Yellow (0.010). In the y-axis title “NF-kappaBn”, the “n” stands for nuclear. (TIFF) [file pone.0027774.s003.tiff]

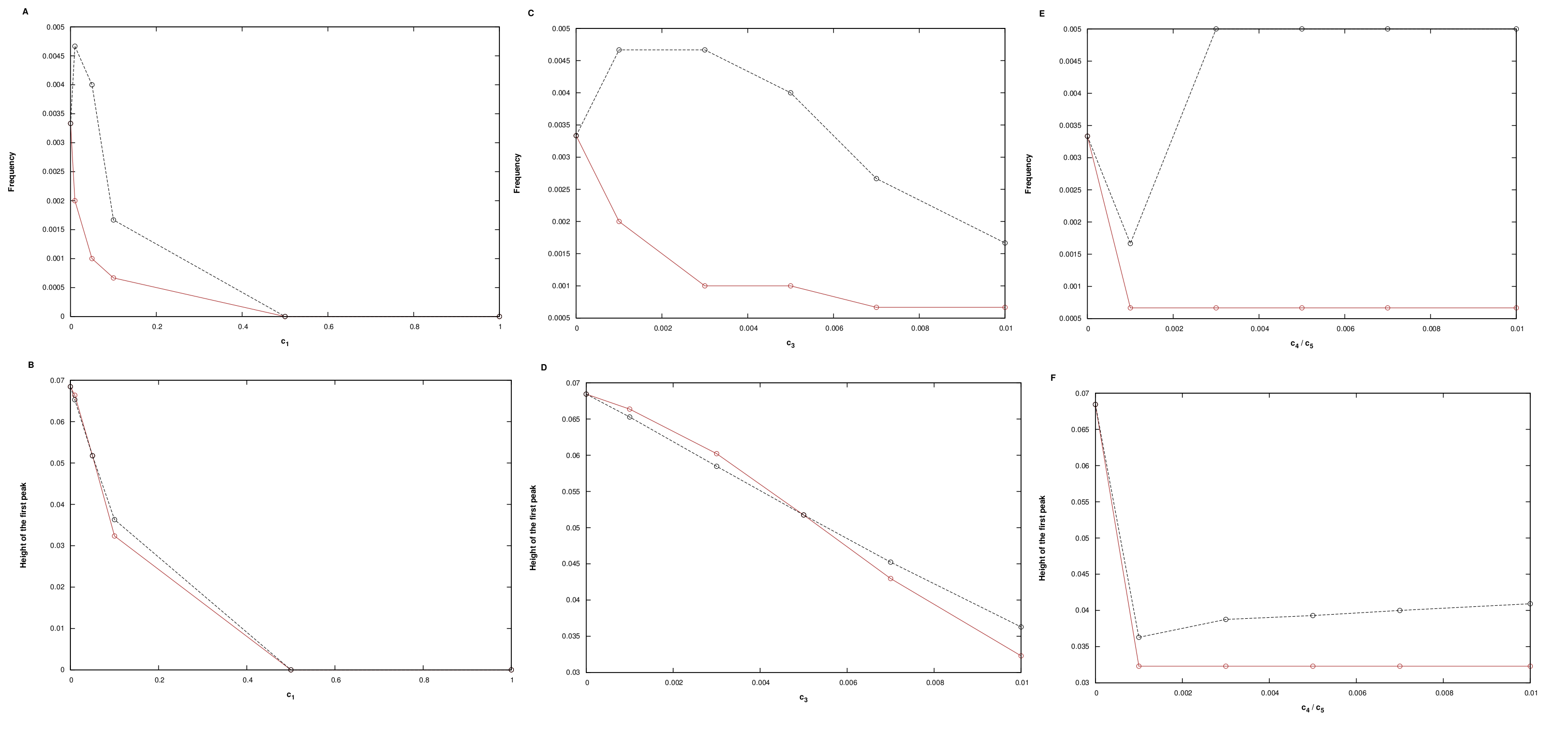

Supplement: Figure S4 — Quantification of the effect of miRNA targeting the IRAK1/TRAF6 complex in the knockout type. When only c1 is varied: A) In terms of alteration in frequency B) In terms of alteration in the height of the first peak. When only c3 is varied: C) In terms of alteration in frequency D) In terms of alteration in the height of the first peak. When only c4 and c5 are varied in Case 1 and Case 2 respectively: E) In terms of alteration in frequency F) In terms of alteration in the height of the first peak. The x-axis signifies the variation of the parameter individually, keeping the other two constant as given in S 3. The Brown and Black lines represent Cases 1 and 2 respectively. (TIFF) [file pone.0027774.s004.tiff]

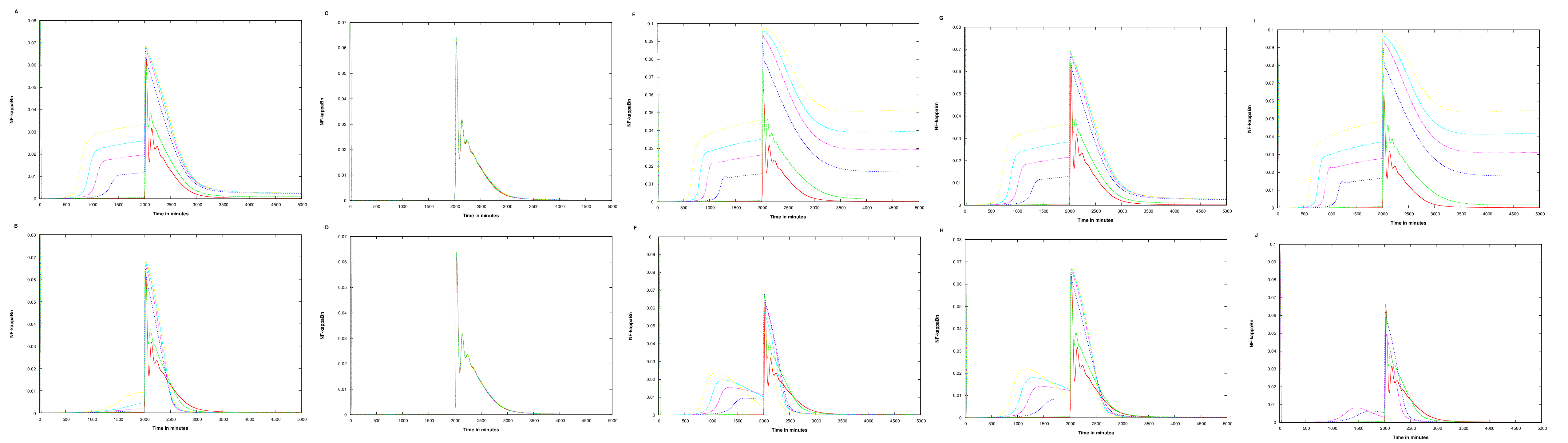

Supplement: Figure S5 — The biphasic plot gets distorted when the miRNA target is: Only IκBα: A) Case 1 B) Case 2. Only IκBε: C) Case 1 D) Case 2. Both IκBα and IκBβ: E) Case 1 F) Case 2. Both IκBα and IκBε: G) Case 1 H) Case 2. All the three IκB's (IκBα, IκBβ, IκBε): I) Case 1 J) Case 2. In the y-axis title “NF-kappaBn”, the “n” stands for nuclear. (TIFF) [file pone.0027774.s005.tiff]
